# Next-Generation Manufacturing Protocols Enriching T_SCM_ CAR T Cells Can Overcome Disease-Specific T Cell Defects in Cancer Patients

**DOI:** 10.3389/fimmu.2020.01217

**Published:** 2020-06-19

**Authors:** Silvia Arcangeli, Laura Falcone, Barbara Camisa, Federica De Girardi, Marta Biondi, Fabio Giglio, Fabio Ciceri, Chiara Bonini, Attilio Bondanza, Monica Casucci

**Affiliations:** ^1^Innovative Immunotherapies Unit, Division of Immunology, Transplantation and Infectious Diseases, IRCCS San Raffaele Scientific Institute, Milan, Italy; ^2^Experimental Hematology Unit, Division of Immunology, Transplantation and Infectious Diseases, IRCCS San Raffaele Scientific Institute, Milan, Italy; ^3^Hematology and Hematopoietic Stem Cell Transplantation Unit, IRCCS San Raffaele Scientific Institute, Milan, Italy; ^4^Vita-Salute San Raffaele University, Milan, Italy

**Keywords:** CAR T, CAR T cell manufacturing, CAR T cell fitness, CAR design, patient samples, B-ALL and PDAC

## Abstract

Chimeric antigen receptor (CAR) T cell expansion and persistence emerged as key efficacy determinants in cancer patients. These features are typical of early-memory T cells, which can be enriched with specific manufacturing procedures, providing signal one and signal two in the proper steric conformation and in the presence of homeostatic cytokines. In this project, we exploited our expertise with paramagnetic beads and IL-7/IL-15 to develop an optimized protocol for CAR T cell production based on reagents, including a polymeric nanomatrix, which are compatible with automated manufacturing *via* the CliniMACS Prodigy. We found that both procedures generate similar CAR T cell products, highly enriched of stem cell memory T cells (T_SCM_) and equally effective in counteracting tumor growth in xenograft mouse models. Most importantly, the optimized protocol was able to expand CAR T_SCM_ from B-cell acute lymphoblastic leukemia (B-ALL) patients, which in origin were highly enriched of late-memory and exhausted T cells. Notably, CAR T cells derived from B-ALL patients proved to be as efficient as healthy donor-derived CAR T cells in mediating profound and prolonged anti-tumor responses in xenograft mouse models. On the contrary, the protocol failed to expand fully functional CAR T_SCM_ from patients with pancreatic ductal adenocarcinoma, suggesting that patient-specific factors may profoundly affect intrinsic T cell quality. Finally, by retrospective analysis of *in vivo* data, we observed that the proportion of T_SCM_ in the final CAR T cell product positively correlated with *in vivo* expansion, which in turn proved to be crucial for achieving long-term remissions. Collectively, our data indicate that next-generation manufacturing protocols can overcome initial T cell defects, resulting in T_SCM_-enriched CAR T cell products qualitatively equivalent to the ones generated from healthy donors. However, this positive effect may be decreased in specific conditions, for which the development of further improved protocols and novel strategies might be highly beneficial.

## Introduction

Chimeric antigen receptor (CAR) T cell therapy for B-cell tumors has so far gained impressive clinical results, leading to unprecedentedly high complete remission rates in patients resistant to standard treatments (1–4).

However, frequent relapses in treated patients, together with inability to achieve complete remission in other disease types (4–7), underline that additional efforts at the preclinical level are needed to improve the efficacy of this innovative therapeutic strategy (4, 8, 9). Recent clinical experience has clearly indicated that CAR T cells have to accomplish several features to sustain an effective and long-lasting anti-tumor response. In particular, CAR T cell expansion immediately after infusion and long-term persistence after initial tumor control represent crucial efficacy determinants (10). It has become increasingly evident that these properties can be enhanced by enriching early memory CAR T cell subsets, e.g., stem cell memory (T_SCM_) and central memory (T_CM_) T cells, by reducing the expression of inhibitory and exhaustion markers and by activating metabolic programs that foster oxidative phosphorylation and fatty acid oxidation (4, 11). In addition, especially when dealing with solid tumors, CAR T cells need to traffic to the tumor sites, recognize tumor cells and expand in an extremely immunosuppressive environment (9, 12). Therefore, proper attention should be dedicated to the creation of CAR T cell products capable of facing multiple challenges at a time, depending on tumor context and disease-specific factors. Hence, the capability of manufacturing protocols to shape the final CAR T cell product so that it could succeed in all these aspects currently represents one of the major goals of pre-clinical research in the field.

T cell fitness can be influenced by multiple factors, such as patients' features, like disease histology, age, prior treatments and the presence of a hostile microenvironment, which can compromise proper functionality of the T cell compartment (4, 13, 14). This is the case of chronic lymphoblastic leukemia (CLL), where baseline T cell dysfunction seems to be the primary cause of resistance to CAR T cell therapy, which proved effective in only 15–30% of patients (3, 5, 6) if not combined with other drugs, such as ibrutinib, which significantly increased response to treatment (15). In particular, it has been reported that T cells from non-responding CLL patients, either contained in the apheresis or in the final CAR T cell product, have a metabolic, phenotypic and transcriptomic signature associated with T cell exhaustion and late memory (11). Moreover, a reduction in the initial naïve T cell (T_N_) content has been reported in several tumor contexts already at the diagnosis and even more pronounced after repeated chemotherapy cycles, resulting in the failure to generate productive CAR T cell formulations (16). Accordingly, ~10–20% of therapeutic failures are still due to hurdles in the manufacturing process (17), with CAR T cell productions still skewed toward those patients displaying an absolute lymphocyte count around the physiological range, i.e. ~1200 cells/uL (18, 19).

In keeping with this, procedures for T cell manufacturing have evolved over time in order to generate CAR T cells highly enriched in T_SCM_ and T_CM_ and endowed with improved *in vivo* fitness. This goal has been accomplished by providing both signal one and signal two in the proper steric conformation, e.g., through cell-sized beads (20) or polymeric nanomatrices (21), and in the presence of homeostatic cytokines, such as IL-7 and IL-15. More recently, it has been described that the activation of pre-selected T_N_ cells in the presence of specific cytokines (22, 23), Wnt agonists (13) or antioxidant molecules (24) could further improve the quality of the final T cell product (10).

Presently, standardized protocols for CAR T cell manufacturing are still missing, with the overall processes being extremely complex, as comprising multiple handling steps, each one capable of causing operator errors, compromising the overall reproducibility. On the contrary, efficient clinical translation of CAR T cell therapies would require the development of optimized and automated protocols, which allow to reduce costs and errors, while increasing standardization and reproducibility. In this regard, the TransAct T cell activation reagent, a polymeric nanomatrix agonist for CD3 and CD28, has been recently reported to be compliant to good manufacturing procedures (GMP) guidelines and compatible with the CliniMACS Prodigy device (17, 25), which permits the enrichment of cellular products under a closed and standardized system (17).

In this manuscript, we exploited the best-performing T cell activation protocol developed in our institution (20, 22, 23, 26), which is based on αCD3/αCD28 paramagnetic beads and IL-7/IL-15, for setting up a nanomatrix-based procedure compliant with automated CAR T cell manufacturing. We investigated the effect of this protocol on different T cell sources, either derived from healthy donors (HDs) or patients suffering from B-cell acute lymphoblastic leukemia (B-ALL) and pancreatic ductal adenocarcinoma (PDAC). These analyses revealed that CAR T cells generated with nanomatrix and paramagnetic beads are comparable and equally enriched in T_SCM_, whose frequency in the manufactured product was found to positively correlate with anti-tumor activity in xenograft mouse models. Moreover, we observed that T cells derived from B-ALL and PDAC patients are differently responsive to the manufacturing procedure, indicating the existence of intrinsic T cell defects that, depending on tumor of origin, patients' age and previous treatments, require or not the development of additional strategies to be efficiently overcome.

## Materials and Methods

### Primary T Cell Culture, Transduction and Stimulation

Peripheral blood mononuclear cells (PBMCs) were isolated by density gradient centrifugation (Lymphoprep, Sentinel Diagnostics). Peripheral blood lymphocytes (PBLs) were sorted with the CD4 and CD8 Isolation Kits (Miltenyi Biotec). PBLs were activated with αCD3/αCD28 beads (Dynabeads, Thermofisher CTS™ Cell Therapy Systems) or αCD3/αCD28 nanomatrix (T-Cell TransAct Reagent, Miltenyi Biotec) according to the following procedures. α*CD3/*α*CD28 beads*: PBLs were stimulated at the 3:1 bead:cell ratio and lentivirally (LV)-transduced at day 2 (multiplicity of infection, MOI: 5). Beads were removed by magnetic separation at day 6 and cells were expanded till day 14, when they were cryopreserved until use. α*CD3/*α*CD28 nanomatrix*: PBLs were stimulated with the nanomatrix according to manufacturing instructions and LV-transduced at day 1 (MOI: 5). The nanomatrix was removed by centrifugation at day 2 and cells expanded till day 14, when they were cryopreserved until use. In both protocols, T cells were cultured in the TexMACS medium (Miltenyi Biotec) supplemented with 1% penicillin/streptomycin (100 U/ml and 0,1 mg/ml, Euroclone), IL-7 (25 U/ml, Miltenyi Biotech) and IL-15 (50 U/ml, Miltenyi Biotec) in the presence of 3% fetal bovine serum (FBS, Carlo Erba). Only experiments showed in Supplementary Figure 1 were performed in serum-free conditions.

Buffy coats from healthy donors were obtained after written informed consent. All patients signed informed consent forms approved by the Ospedale San Raffaele Ethics Committee, in accordance with the declaration of Helsinki. B-ALL samples were selected on the basis of the disease classification (type B), a leukemic blast content inferior to 50% in peripheral blood (PB) and no prior transplantation. Patients' characteristics are summarized in Tables 1, 2.

**Table 1:** List of B-cell acute lymphoblastic leukemia (B-ALL) patient samples.

**Patient^**#**^**	**Tumor**	**Age**	**Sex**	**CT cycles**	**Administered drugs**
1	B-ALL	36	M	4	Metotrexate-Cytarabine
2	B-ALL	18	M	1	Vincristine-Idarubicin- Dexamethasone-Asparaginase
3	B-ALL	19	M	6	Vincristine-Idarubicin-Dexamethasone- Cyclophosphamide- Cytarabine-6Mercaptopurine
4	B-ALL	20	F	7	Clofarabine-Cyclophosphamide-Etoposide
5	B-ALL	62	M	6	Metotrexate-Cytarabine
6	B-ALL	21	F	9	Mitoxantrone-Cytarabine
7	B-ALL	37	M	7	Metotrexate-Cytarabine
8	B-ALL	55	F	11	Blinatumomab
9	B-ALL	29	M	5	Metotrexate-Asparaginase- 6Mercaptopurine
10	B-ALL Ph+	54	M	8	Dasatinib-Vincristine-Idarubicin- Dexamethasone-Prednisone
11	B-ALL Ph+	58	M	8	Metotrexate-Cytarabine
12	B-ALL Ph+	43	M	3	Imatinib-Metotrexate-Cytarabine
13	B-ALL Ph+	35	M	3	Imatinib-Metotrexate-Cytarabine

*B-ALL Ph+, B-ALL positive for Philadelphia chromosome translocation; CT, chemotherapy*.

**Table 2:** List of pancreatic ductal adenocarcinoma (PDAC) patient samples.

**Patient^**#**^**	**Tumor**	**Age**	**Sex**	**Last treatment**
1	PDAC	76	M	none
2	PDAC	N/A	N/A	none
3	PDAC	57	M	none
4	PDAC	N/A	N/A	none
5	PDAC	66	F	none
6	PDAC	47	M	none
7	PDAC	73	M	none

*N/A, not available*.

### Cell Lines

Cell lines were cultured in RPMI 1640 (Lonza) supplemented with 10% FBS (Carlo Erba), 1% penicillin (100 U/ml, Euroclone)/streptomycin (0,1 mg/ml, Euroclone) and 1% L-glutamine (2 mM, Euroclone).

For *in vivo* experiments, RAJI, NALM-6 and BxPC-3 cell lines were transduced with a bidirectional lentiviral vector encoding for the secreted Gaussia luciferase Lucia (Invivogen) and the LNGFR selection marker, which allowed the isolation of transduced cells. The lentiviral bidirectional construct was kindly provided by Prof. Luigi Naldini. The Gaussia Luciferase is actively secreted by cells and detectable in the blood allowing the easy monitoring of tumor progression in mice (27).

### Vector Constructs

The expression cassettes for both the CD19 and EGFR CARs are comprised in bidirectional lentiviral vectors provided by Miltenyi Biotech in the context of the European project horizon 2020-CARAT. The vectors contain the second-generation CAR linked to the selection marker NGFR by means of a sequence encoding a 2A element. The different CARs are composed by an extracellular domain derived from the single chain fragment variable (scFv) of a monoclonal antibody directed against the CD19 (FMC63) or EGFR (high affinity: Cetuximab, low affinity: Nimotuzumab) antigens, linked to the CD3z chain of the TCR complex by means of a CD8 spacer and transmembrane domain, together with the 4-1BB co-stimulatory domain.

### Co-culture Assays

CAR T cells and un-transduced (CTRL) effector cells were co-cultured at different effector to target (E:T) ratios with tumor cells (CD19^+^ RAJI, NALM-6 and BV173 cells; CD19^−^ MM.1S cells; EGFR^+^ BxPC3). After 4 days, CAR T cells and target cells were discriminated and counted by fluorescence-activated cell sorting (FACS) analysis, using cell-specific markers and Flow-Count Fluorospheres (BeckmanCoulter).

The elimination index (EI) was calculated as follows: 1–(number of residual target cells in presence of CAR T cells/number of target cells in presence of control T cells). Supernatants were collected after 24 h of co-culture to analyze cytokine release with the Th1/Th2 LEGENDplex assay (Biolegend), according to manufacturer's instructions. Data were analyzed with the software provided by the kit and subsequently with Prism software 8.1.1 (GraphPad).

### *In vivo* Experiments

All mouse experiments were approved by the institutional animal care and use committee (IACUC) of San Raffaele University Hospital and Scientific Institute and by the Italian Governmental Institute of Health (Rome, Italy).

Six to 8-week-old female or male NOD.Cg-Prkdcscid IL-2rgtm1Wjl/SzJ (NSG) mice were infused intravenously with 0.5 × 10^6^ Lucia^+^/LNGFR^+^ RAJI or Lucia^+^/LNGFR^+^ NALM-6 cells and, after 7 and 4 days, respectively, treated with 4 × 10^6^ and 3 × 10^6^ CD19 CAR T cells from HDs, patients or CTRL. The same experimental setting was used by injecting 0.5 × 10^6^ Lucia^+^/LNGFR^+^ BxPC3 cells. In this case, NSG mice were treated with 5 × 10^6^ CTRL and EGFR CAR T cells obtained from PDAC patients. Tumor progression was monitored twice a week by bioluminescence, using the QUANTI-Luc detection reagent (InvivoGen) and expressed as relative light units (RLUs), according to the manufacturer instructions. Circulating human T cell counts were measured by FACS using Flow-Count Fluorospheres (BeckmanCoulter). Mice were sacrificed when tumor growth reached the threshold value of 10^6^ RLU for NALM-6 and BxPC3 and 0.5 × 10^5^ for RAJI, or when manifesting signs of suffering.

### Flow Cytometry

T cell basal and post-activation phenotypes were characterized by flow cytometry staining with FITC, PE, PerCP, PeCy7, APC, APC-H7, Pacific Blue, BV510 conjugated antibodies, and analyzed with a FACS Canto II flow cytometer (BD Biosciences). CAR T cells and mouse samples were stained with one or more of the following conjugated monoclonal antibodies: anti-human CD3 PB (Biolegend, clone HIT3a), CD45 BV510 (Biolegend, clone HI30), CD271 PE-Cy7 (Biolegend, clone CD40-1457), CD271 PE (BD, clone C40-1457), CD4 FITC (Biolegend, clone SK3), CD14 APC (Biolegend, clone M5E2), CD19 APC/Cy7 (Biolegend, clone HIB19), HLA-DR APC/Cy7 (Biolegend, clone L243), CD45RA FITC (Biolegend, clone HI100), CD62L APC (Biolegend, clone DREG-56), CD8 PerCP (BD, clone SK1), CD57 APC/Cy7 (Milteny, clone TB03), CD127 PE (Biolegend, clone A019D5), EGFR PE (Biolegend, clone AY13), CD95 (Fas/APO-1) PE-Cy7 (Biolegend, clone DX2), CD279 (PD-1) PE-Cy7 (Biolegend, clone EH12.1), TIM-3 Alexa Fluor 488 (Biolegend, clone F38-2E2), and anti-mouse CD45 PerCP (Biolegend, clone 30-F11). 7-Aminoactinomicin D (7-AAD, Biolegend) and DAPI were used to discriminate viable and non-viable cells.

All data were analyzed with the Flow Jo_V10 software (Tree Star Inc.).

### Statistical Analyses

Statistical analyses were performed with Prism Software 8.1.1 (GraphPad). Data are shown as Mean ± SEM with at least *n* = 3 replicates. Datasets were analyzed with paired/unpaired Student's *t*-test or one way/two-way ANOVA tests, depending on the experimental design considered. Differences with a *P* value < 0.05 were considered as statistically significant.

## Results

### Nanomatrix- and Paramagnetic Beads-Based Protocols Generate Similar CAR T Cell Products

In order to verify the robustness of nanomatrix-based CAR T cell manufacturing, we compared it with the best-performing procedure developed in our laboratory, based on activation with paramagnetic beads and culture with homeostatic cytokines (20, 23, 26, 28). While in one platform the αCD3 and αCD28 antibodies are covalently coupled to cell-size magnetic beads, in the other they are rather embedded in a small, polymeric and biodegradable nanomatrix, leading thus to an easier and faster removal passage compatible with CliniMACS Prodigy device (29). Briefly, peripheral blood (PB) T lymphocytes from HDs were stimulated with either the nanomatrix or paramagnetic beads, transduced with a lentiviral vector encoding for a 4-1BB co-stimulated CD19 CAR (CD19.BBz CAR), and cultured in the presence of low concentrations of IL-7 and IL-15. To get close to GMP-grade manufacturing, T cells were kept in culture in serum-free TexMACS medium. Interestingly, both T cell populations expanded similarly up to day 14, while at later times points CAR T cells generated with paramagnetic beads acquired a significant proliferative advantage (Supplementary Figure 1A). Of notice, transduction efficiency was slightly but significantly higher after stimulation with paramagnetic beads than with the nanomatrix. This discrepancy could be explained by a different activation kinetic, which was anticipated and milder in the case of the nanomatrix compared to paramagnetic beads (Supplementary Figure 1B). Moreover, we observed that the addition of a small amount of FBS significantly increased fold expansion of CAR T cells generated with the nanomatrix (Supplementary Figure 1C). For these reasons, we modified both the manufacturing procedures by implementing 3% serum to the TexMACS medium and by anticipating lentiviral transduction of nanomatrix-activated T cells from day 2 to day 1.

**Figure 1:**
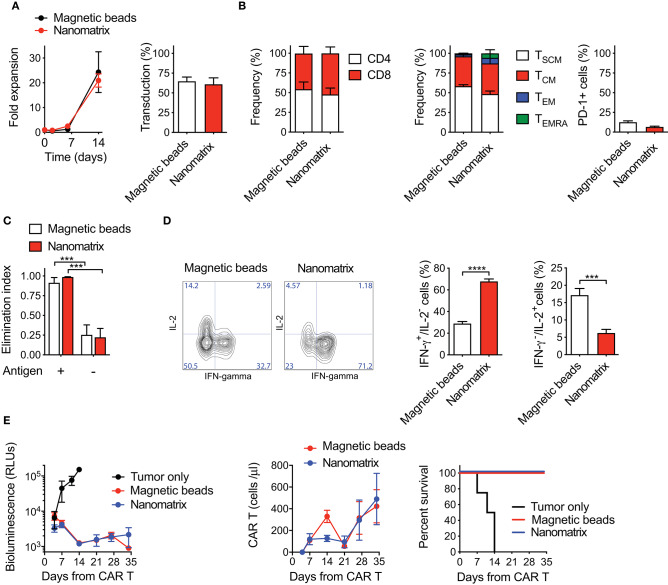
CAR T cells generated with either the nanomatrix or paramagnetic beads are biologically equivalent. **(A)** Analysis of fold expansion in culture and transduction efficiency at day 8 of both nanomatrix- and paramagnetic beads-generated CD19.BBz CAR T cells (*n* = 3). **(B)** Phenotypical characterization of CAR T cells generated with both the nanomatrix and paramagnetic beads protocols at the end of the culture period, illustrating the CD4/CD8 ratio (left, *n* = 5), T cell memory compartments (middle, *n* = 5) and PD-1 expression (right, *n* = 5). T_SCM_: CD45RA^+^CD62L^+^; T_CM_: CD45RA^−^CD62L^+^; T_EM_: CD45RA^−^CD62L^−^; T_EMRA_: CD45RA^+^CD62L^−^. **(C)** Killing assay performed by co-culturing nanomatrix- and paramagnetic beads-generated CAR T cells with CD19^+^ BV-173 cells and CD19^−^ MM.1S cells for 4 days at the 1:5 effector to target (E:T) ratio (*n* = 3). **(D)** Intracellular staining depicting IFN-γ and IL-2 production by nanomatrix- and paramagnetic beads-generated CAR T cells after 24 h stimulation with PMA/Ionomicin. Left panel: representative dot plots. Right panel: mean ± SEM (*n* = 5). **(E)** Left panel: bioluminescence detection of Lucia^+^/NGFR^+^ RAJI systemic growth in NSG mice after treatment with nanomatrix- and paramagnetic beads-generated CAR T cells, together with Mock control. Middle panel: CAR T cell expansion kinetic in the peripheral blood (PB) of RAJI-bearing mice, together with Mock control. Right panel: survival curve of RAJI-bearing mice treated with different CAR T cell populations or Mock control. Data are reported as Kaplan–Meyer survival plots for mice and results of a Mantel–Cox two-sided log-rank test are reported when statistically significant (*n* = 6 mice/group; *n* = 4 mice for Mock control, 1 donor). Except for survival plots, data are reported as the result of mean ±SEM and paired *t*-test and two-way ANOVA statistical analyses are reported when statistically significant (***p < 0.001; ****p < 0.0001).

Under these optimized conditions, CAR T cells generated with the nanomatrix expanded similarly to CAR T cells obtained with paramagnetic beads and achieved comparable transduction efficiencies (Figure 1A). An equivalent CD4/CD8 ratio was reached as well, together with a substantial enrichment of early memory T cell compartments, including both T_SCM_ (CD45RA^+^CD62L^+^CD95^+^) and T_CM_ (CD45RA^−^CD62L^+^) in the absence of relevant PD-1 expression (Figure 1B). When challenged against CD19^+^ targets *in vitro*, both CAR T cell products displayed a similar cytotoxic activity (Figure 1C), even though CAR T cells generated with the nanomatrix featured a higher proportion of IFN-γ^+^/IL-2^−^ cells compared to the ones obtained with paramagnetic beads, pointing toward a stronger effector signature of the formers (Figure 1D). Most importantly, when challenged in NSG mice against CD19^+^ RAJI lymphoma cells, CAR T cells manufactured with either the nanomatrix or paramagnetic beads mediated a comparable anti-tumor activity and displayed similar expansion kinetics, resulting in super-imposable survival curves (Figure 1E).

Collectively, these results indicate that CAR T cells generated with reagents compatible with automated manufacturing via the CliniMACS Prodigy are phenotypically and functionally equivalent to CAR T cells generated with paramagnetic beads.

### Optimized Manufacturing Can Rescue the Phenotype of T Cells Derived From B-ALL, but Not PDAC Patients

Afterwards, we investigated the ability of the nanomatrix-based manufacturing procedure to generate fully functional CAR T cells from patients suffering from B-ALL and PDAC. Patients' characteristics are listed in Tables 1, 2.

**Figure 2:**
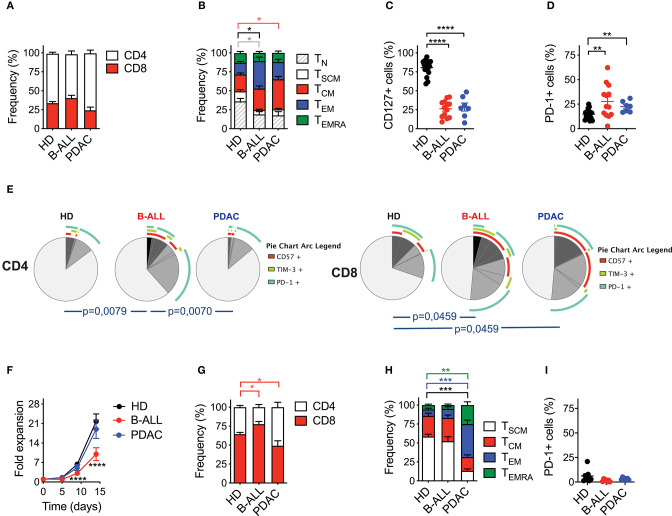
Optimized CAR T cell manufacturing is able to rescue the phenotype of T cells derived from cancer patients in a disease-specific manner. **(A–E)** Phenotypical characterization of healthy donor- (HD), B-cell acute lymphoblastic leukemia- (B-ALL) and pancreatic ductal adenocarcinoma (PDAC)-derived T cells in PB prior activation with the nanomatrix protocol, illustrating **(A)** CD4/CD8 ratio (*n* = 16 for HD; *n* = 12 for B-ALL; *n* = 7 for PDAC), **(B)** T cell memory phenotype (*n* = 7 for HD; *n* = 13 for B-ALL; *n* = 7 for PDAC), T_N_: CD45RA^+^CD62L^+^CD95^−^; T_SCM_: CD45RA^+^CD62L^+^CD95^+^; T_CM_: CD45RA^−^CD62L^+^; T_EM_: CD45RA^−^CD62L^−^; T_EMRA_: CD45RA^+^CD62L^−^, **(C)** CD127 expression (*n* = 16 for HD; *n* = 13 for B-ALL; *n* = 7 for PDAC) and **(D)** PD-1 expression (*n* = 16 for HD; *n* = 13 for B-ALL; *n* = 7 for PDAC). **(E)** Analysis of PB T cell exhaustion by means of SPICE algorithm calculated on both CD4 and CD8 compartments, depicting concomitant expression of inhibitory and senescence markers (PD-1, TIM-3 and CD57, *n* = 7 for HD; *n* = 11 for B-ALL; *n* = 7 for PDAC). **(F)** Fold expansion in culture of HDs and patient-derived T cells after activation with the nanomatrix protocol and transduction with a CD19.BBz CAR (*n* = 10 for HD; *n* = 7 for B-ALL; *n* = 7 for PDAC). **(G–I)** Phenotypical characterization of HDs and patient-derived CD19.BBz CAR T cells after activation with the nanomatrix protocol, illustrating **(G)** the CD4/CD8 ratio, **(H)** T cell memory phenotype and **(I)** PD-1 expression (*n* = 12 for HD; *n* = 7 for B-ALL; *n* = 7 for PDAC). Data are reported as the result of mean ±SEM and paired *t*-test and two-way ANOVA statistical analyses are reported when statistically significant (*p < 0.05, **p < 0.01, ***p < 0.001,****p < 0.0001).

Analysis of T cell composition in starting PB samples revealed a similar CD4/CD8 ratio in patients' T cells, as compared to HDs (Figure 2A). However, patients' T cells featured lower proportions of T_N_ (CD45RA^+^CD62L^+^CD95^−^) and T_SCM_ (CD45RA^+^CD62L^+^CD95^+^) compared to HDs (Figure 2B, Supplementary Figure 2A), together with a lower frequency of T cells expressing the IL-7Rα (CD127) (Figure 2C, Supplementary Figure 2B). Such differentiated signature, typical of patients' T cells as opposed to HDs, was observed in both the CD4 and CD8 compartments (Supplementary Figure 3A). Moreover, cancer patients were characterized by a higher frequency of T cells co-expressing one or more inhibitory receptors and senescence markers, such as TIM-3, PD-1 and CD57 (Figures 2D,E and Supplementary Figures 3B, 4A–C). Interestingly, both CD4^+^ and CD8^+^ cells were phenotypically compromised in B-ALL patients, while the exhaustion signature was limited to the CD8 compartment in PDAC patients.

We then proceeded to assess the responsiveness of different T cell sources to the manufacturing procedure. PB T cells from both HDs and cancer patients were engineered according to the optimized protocol based on the nanomatrix reagent. To exclude potential variability deriving from different CAR constructs and target antigens, all T cell populations, including those from PDAC patients, were transduced with a CD19.BBz CAR. Interestingly, PDAC CAR T cells expanded similarly to HD CAR T cells, while B-ALL CAR T cells expanded less (Figure 2F). As expected, in all groups the CD8 compartment was significantly enriched (Figure 2G). Strikingly, however, while the optimized protocol succeeded in expanding early memory CAR T cells from B-ALL patients, it failed to enrich these subsets in PDAC patients (Figure 2H, Supplementary Figure 5A). Indeed, while more than 80% of B-ALL CAR T cells were either T_SCM_ or T_CM_ (CD45RA^−^CD62L^+^) PDAC CAR T cells were significantly enriched in T_EM_ (CD45RA^−^CD62L^−^) and terminal effectors (CD45RA^+^CD62L^−^). On the other hand, the reduction in the frequency of PD-1^+^ CAR T cells were observed in all conditions (Figure 2I, Supplementary Figure 5B), in line with the application of a protocol known to increase the overall CAR T cell fitness. Importantly, similar observations can be applied to both the CD4 and CD8 compartments (Supplementary Figure 3C).

Taken together, these results suggest that the employment of the nanomatrix-based manufacturing protocol, conceived for enriching early memory T cell subsets and their associated fitness, was able to rescue the phenotype of T cells derived from B-ALL but not PDAC patients, highlighting the existence of disease-intrinsic T cell defects that are differentially responsive to the manufacturing procedure.

### Optimized Manufacturing Can Generate Fully Functional CAR T Cells From B-ALL, but Not PDAC Patients

**Figure 3:**
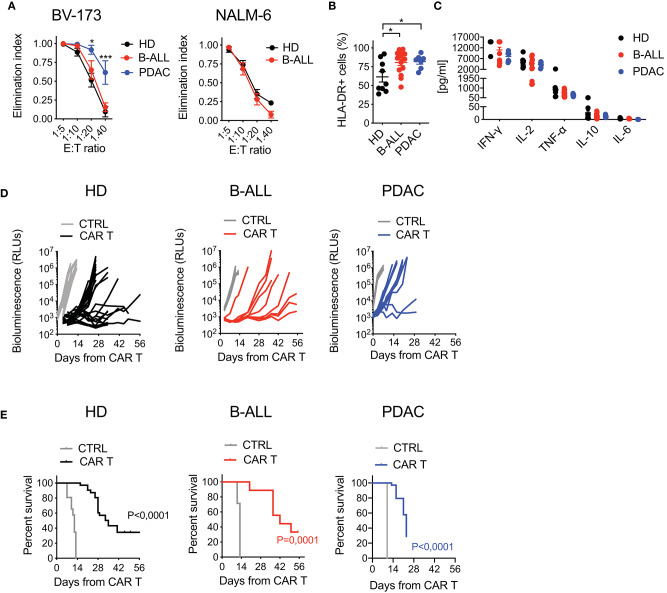
Optimized CAR T cell manufacturing is able to generate fully functional CAR T cells from cancer patients in a disease-specific manner. **(A)** Killing assays performed by co-culturing HD- and patient-derived CD19.BBz CAR T cells with CD19^+^ targets (BV-173 and NALM-6 cell lines) for 4 days at different E:T ratios (*n* = 7 donors/group). **(B)** Analysis of CAR T cell activation, by means of HLA-DR expression, after co-culture with CD19^+^ target cells for 4 days at the 1:10 E:T ratio (*n* = 9 for HD; *n* = 7 for B-ALL; *n* = 7 for PDAC). **(C)** Quantification of pro-inflammatory cytokine production after 24 h co-culture of CAR T cells with CD19^+^ targets at the 1:10 E:T ratio (*n* = 9 for HD; *n* = 7 for B-ALL; *n* = 7 for PDAC). **(D)** Bioluminescence detection of Lucia^+^/LNGFR^+^ NALM-6 systemic growth in NSG mice after treatment with 3 × 10^6^ HD- and patient-derived CD19.BBz CAR T cells, together with Mock control (*n* = 26 mice for HD, 8 donors, and *n* = 19 mice for Mock control, 6 donors; *n* = 9 mice for B-ALL and *n* = 7 mice for Mock control, 3 patients: #5, #8, #9 in Table 1; *n* = 12 mice for PDAC and *n* = 3 mice for Mock control, 3 patients: #1, #2, #3 in Table 2). Data are reported as the result of mean ±SEM and paired *t*-test and two-way ANOVA statistical analyses are reported when statistically significant (*p < 0.05, ***p < 0.001). **(E)** Survival curves of Lucia^+^/LNGFR^+^ NALM-6 leukemia bearing mice treated with HD- and patient-derived CAR T cells, together with Mock control (*n* = 26 mice for HD, 8 donors, and *n* = 19 mice for Mock control, 6 donors; *n* = 9 mice for B-ALL and *n* = 7 mice for Mock control, 3 patients: #5, #8, #9 in Table 1; *n* = 12 mice for PDAC and *n* = 3 mice for Mock control, 3 patients: #1, #2, #3 in Table 2). Data are reported as Kaplan–Meyer survival plots for mice and result of a Mantel–Cox two-sided log-rank test are reported when statistically significant.

To test the functional profile of CAR T cells generated from cancer patients and HDs, we challenged them in co-culture experiments against CD19^+^ tumor cells. While B-ALL and HD CAR T cells performed similarly, the lytic activity of PDAC CAR T cells was significantly higher, possibly reflecting the more differentiated phenotype (Figure 3A). Interestingly, CAR T cell activation in response to target antigen encounter was superior for patients' CAR T cells compared to HDs, as observed by increased HLA-DR expression levels in these conditions (Figure 3B). Conversely, the production of pro-inflammatory cytokines was similar among all CAR T cell products (Figure 3C). The specificity of CAR T cell targeting was confirmed by lack of lysis, milder activation and minimal cytokine production in response to CD19^−^ tumor cells (Supplementary Figures 6A,B).

Next, in order to assess *in vivo* activity in the B-ALL setting, we challenged the different CAR T cell populations in NSG mice infused with NALM-6 leukemia cells. Importantly, B-ALL CAR T cells were as effective as HD CAR T cells in mediating anti-tumor responses, significantly increasing the survival of treated mice. On the contrary, PDAC CAR T cells failed to efficiently counteract leukemia growth, resulting in only a mild survival prolongation (Figures 3D,E).

**Figure 4:**
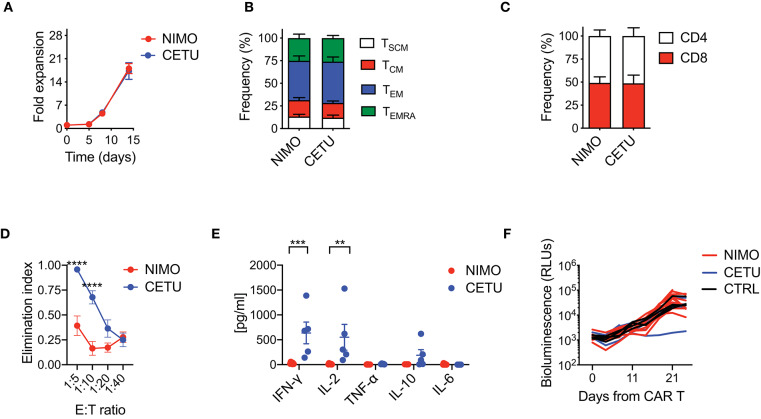
Optimized CAR T cell manufacturing fails to generate EGFR CAR T cells effective against pancreatic cancer. **(A)** Fold expansion in culture of PDAC-derived T cells after activation with the nanomatrix protocol and transduction with EGFR.BBz CARs displaying high (CETU) and low (NIMO) affinity toward EGFR (*n* = 5 for NIMO; *n* = 7 for CETU). **(B,C)** Phenotypical characterization of PDAC-derived EGFR.BBz CAR T cells at the end of the culture period, showing T cell memory compartments and CD4/CD8 ratio (*n* = 5 for NIMO; *n* = 7 for CETU). T_SCM_: CD45RA^+^CD62L^+^CD95^+^; T_CM_: CD45RA^−^CD62L^+^; T_EM_: CD45RA^−^CD62L^−^; T_EMRA_: CD45RA^+^CD62L^−^. **(D)** Killing assays performed by co-culturing PDAC-derived EGFR.BBz CAR T cells with BxPC3 EGFR^+^ target cells for 4 days at different E:T ratios (*n* = 5 for NIMO; *n* = 7 for CETU). **(E)** Quantification of pro-inflammatory cytokine production after 24 h co-culture of CAR T cells with BxPC3 EGFR^+^ cells at the 1:10 E:T ratio (*n* = 5 for NIMO; *n* = 7 for CETU). **(F)** Bioluminescence detection of Lucia^+^/LNGFR^+^ BxPC3 systemic growth in NSG mice after treatment with 5 × 10^6^ EGFR.BBz CAR T cells, together with Mock control, respectively (*n* = 10 mice for NIMO, 3 patients: #2, #3, #7 in Table 2; *n* = 4 mice for CETU, 3 patients: #1, #3, #7 in Table 2; *n* = 4 mice for Mock control, patient #3 in Table 2). Data are reported as the result of mean ±SEM and paired *t*-test and two-way ANOVA statistical analyses are reported when statistically significant (**p < 0.01, ***p < 0.001; ****p < 0.0001).

With the aim of confirming hypo-responsiveness of PDAC CAR T cells with other CAR specificities and in the pancreatic context, we generated two EGFR-targeting CARs incorporating the 4-1BB co-stimulatory domain and including either the high or low affinity scFv from cetuximab (CETU) or nimotuzumab (NIMO), respectively (30). Even in this setting, we confirmed that the optimized manufacturing procedure was not sufficient *per se* for rescuing the phenotype and functionality of CAR T cells derived from PDAC patients. Similar to what observed with the CD19 CAR, EGFR CAR T cells generated from PDAC patients significantly expanded in culture (Figure 4A) and were highly enriched in effectors while devoid of early memory subpopulations (Figure 4B), with also a similar proportion of CD4 and CD8 subsets (Figure 4C). Interestingly, while CETU CAR T cells exerted cytotoxic activity *in vitro*, NIMO CAR T cells did not (Figure 4D), suggesting that BxPC3 did not express EGFR at sufficient levels to be recognized by low-affinity CAR T cells (30, 31). This behavior was also confirmed in terms of cytokine release (Figure 4E). Finally, we observed that neither CAR T cell condition efficiently counteracted the growth of BxPC3 pancreatic cells in NSG mice (Figure 4F), supporting the notion that the activity of CAR T cells from PDAC patients is hampered by intrinsic functional defects.

Overall, these results suggest that in B-ALL optimized manufacturing procedures have the potential to overcome initial T cell defects, generating completely functional CAR T cells. Conversely, in PDAC additional improvements in the manufacturing procedures or combination with other therapeutics is needed to ameliorate the final outcome of CAR T cell therapy.

### The Proportion of T_SCM_ Cells in the Final CAR T Cell Product Positively Correlates With *in vivo* Efficacy

**Figure 5:**
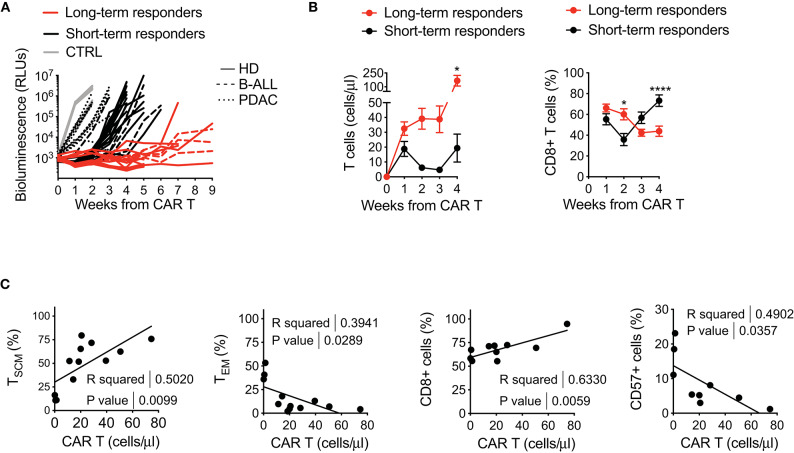
Retrospective correlation of CAR T cell features with therapeutic outcomes *in vivo*. **(A)** Bioluminescence detection of Lucia^+^/LNGFR^+^ NALM-6 systemic growth in NSG mice after treatment with 3 × 10^6^ CD19.BBz CAR T cells of HD- and patient-derived origin, retrospectively clustered as short- and long-term responders (*n* = 18 for long-term responders, red lines; *n* = 29 for short-term responders, black lines. More in detail: *n* = 26 mice for HD, 8 donors, continuous lines; *n* = 9 mice for B-ALL, 3 patients: #5, #8, #9 in Table 1, dashed lines; *n* = 12 mice for PDAC, 3 patients: #1, #2, #3 in Table 2, dotted lines; *n* = 10 mice for Mock control, 3 donors, gray lines). Data are represented as single lines identifying individual mice treated with CD19.BBz CAR T cells of HD- and patient-derived origin and differently clustered on the basis of CAR T cell anti-leukemia activity. **(B)** Left panel: analysis of CD19.BBz CAR T cell expansion kinetic (cell count/μL) of short- and long-term responders in the peripheral blood of Lucia^+^/LNGFR^+^ NALM-6 bearing mice; Right panel: frequency of CD8^+^ CAR T cells during *in vivo* expansion. Data are reported as the result of mean ± SEM and two-way ANOVA statistical analyses are reported when statistically significant (*p < 0.05; ****p < 0.0001). **(C)** Relationships between CAR T cell phenotype and *in vivo* growth after leukemia encounter, correlating the initial proportion of T_SCM_, T_EM_, CD8^+^ and CD57^+^ CAR T cells with *in vivo* expansion levels at day 7 (*n* = 12 for T_SCM_, T_EM_, CD8 and CD57). *R* and *P*-values for each correlation are reported.

Intrigued by the idea of better dissecting the features accounting for long-lasting CAR T cell anti-tumor responses, we retrospectively correlated CAR T cell features with therapeutic outcomes *in vivo*, collecting all data from leukemia-bearing mice treated with CD19 CAR T cells derived from HDs, B-ALL and PDAC patients. By doing this, it was possible to clearly identify two cohorts of short-term and long-term responders, according to the duration of remission after CAR T cell treatment (Figure 5A). Specific analysis of these cohorts revealed predominant differences between the two conditions, with long-term responders relying on superior CAR T cell expansion and higher frequencies of CD8^+^ CAR T cells soon after infusion, followed by expansion of CD4^+^ CAR T cells, as compared to short-term responders (Figure 5B).

The capability of CAR T cells to exert these functions is probably the result of intrinsic features of the final CAR T cell product. Therefore, in order to determine if a defined phenotype *in vitro* could predict a specific behavior *in vivo*, we sought to correlate this aspect with CAR T cell expansion in mice. Of relevance, the frequency of T_SCM_ in the final CAR T cell product positively correlated with CAR T cell expansion *in vivo*, supporting the notion that enriching early memory populations is a key requirement for a successful CAR T cell therapy (Figure 5C). In parallel, also higher frequencies of CD8^+^ CAR T cells were found to correlate with CAR T cell expansion *in vivo*, possibly reflecting the fact that T_SCM_ are preferentially enriched in this T cell subset. On the contrary, the presence of high T_EM_ levels, as well as higher frequency of CD57^+^ cells in the final CAR T cell composition negatively correlated with CAR T cell expansion *in vivo*.

These results confirm clinical evidences that the final therapeutic outcome strictly relies on high CAR T cell expansion after infusion and on a specific CD4/CD8 ratio over time (10, 32). Moreover, we observed that the frequency of T_SCM_ and CD57^+^ cells in the final CAR T cell product can be potentially exploited as predictive positive and negative biomarkers for CAR T cell expansion and anti-tumor efficacy *in vivo*.

## Discussion

So far, retrospective analyses on overall response rates in patients receiving CAR T cell therapy pointed out that CAR T cell fitness is a crucial aspect for gaining therapeutic success, regardless of the tumor context (4, 10). Frequently, however, the application of non-optimized manufacturing procedures failed to generate high-quality CAR T cell products, limiting the achievement of long-lasting anti-tumor responses. Moreover, long and individualized manufacturing is not always compatible for patients with high proliferative diseases and advanced stage cancers, due to the possibility of further progressing during the handling procedure (1, 8, 33, 34). Finally, as a result of limited scalability, high complexity and lack of standardization, the costs associated with the overall process still remain very high, limiting the applicability to a wide number of patients.

It has been previously reported that stimulation with αCD3/αCD28 paramagnetic beads and IL-7/IL-15 proved optimal to enrich early memory CAR T cells, especially T_SCM_, which are endowed with improved ability to expand and persist *in vivo*, becoming particularly attractive for adoptive immunotherapeutic approaches (20, 22, 23, 26, 28). Currently, the CliniMACS Prodigy is the only technology worldwide for the clinical enrichment of cellular products within a closed system (17, 25). As a result, this device offers the unique opportunity to increase standardization and reduce the costs of CAR T cell manufacturing, by diminishing the need for highly experienced personnel and by mitigating clean room requirements. In this work, we proved that CAR T cell products generated with Prodigy-compliant reagents, including the TransAct nanomatrix, are comparable to those manufactured with paramagnetic beads and IL-7/IL-15. Indeed, both protocols proved capable to enrich CAR T cells with a stem memory phenotype, devoid of inhibitory receptors and able to mediate profound anti-tumor responses in xenograft mouse models.

To date, CAR T cell therapy was mainly applied in the autologous setting. However, autologous CAR T cell formulations suffer from several hurdles, which limit the number of patients who can effectively benefit from this therapeutic approach. First, due to a sequela of prior treatments, including chemotherapy, most patients are lymphopenic, challenging the collection of sufficient T cell numbers (8). In addition, reduced frequency of T_N_ has been reported in cancer patients, either at diagnosis and after chemotherapy cycles, resulting in poor performing CAR T cells and production failures (16, 35). Finally, having lived in a tumor-bearing host may have significantly compromised the fitness of patient-derived T cells, resulting in poor CAR T cell performances *in vivo* (4, 8, 9, 36). In this work, we reported initial T cell defects in both B-ALL and PDAC patients, as compared to HDs, including a reduced frequency of T_N_ and T_SCM_, as well as a clear signature of T cell exhaustion, characterized by high expression of inhibitory receptors and senescence markers. Strikingly, however, while in B-ALL the manufacturing procedure was able to rescue initial dysfunctions, expanding T_SCM_ CAR T cells that performed similarly to those from HDs, CAR T cells from PDAC patients were preferentially T_EM_ and failed to exert significant anti-tumor activity *in vivo*, using different CAR designs and specificities. Unfortunately, the small size of our patients' cohorts impedes to draw definitive conclusions on the reasons accounting for what observed. Reasonably, multiple factors could have been involved.

It is known that patients' age *per se* can shape T cell differentiation and senescence, resulting in an overall reduction in lymphocyte collection efficiencies and T cell fitness (14, 37). Although CAR T cell therapy in B-ALL has offered spectacular promise in children and young adults, definitive data on its effectiveness in older individuals are still awaited (38–40). Of notice, our analysis revealed that, despite initial T cell defects, the optimized manufacturing procedure succeeded in generating fully functional CAR T cell products from adult individuals suffering from B-ALL (mean age: 37, range: 18–58). Of relevance, patients with PDAC were much older (mean age: 64, range: 47–76), possibly contributing to the disappointing results obtained with PDAC samples.

Previous studies have indicated that chemotherapy-related depletion of early lineages, especially T_N_, decrease the rate of successful *ex vivo* stimulation responses (16, 35). Notably, even though heavily pretreated and T_N_-deficient, our cohort of B-ALL patients successfully responded to the optimized manufacturing procedure, supporting the notion that proper activation in the presence of IL-7 and IL-15 can rescue initial T cell defects (35). Conversely, our PDAC patients were chemotherapy-naïve, supporting a prominent impact of the tumor itself. Despite the lack of post-treatment PDAC samples to use as comparison, this hypothesis finds some clues in the literature. Indeed, it has been described that CD8^+^ T cells infiltrating solid tumors exist in two dysfunctional states, either reversible or permanent, depending on the respective chromatin arrangements (41). Whether this signature is equally ascribed to solid tumor-derived T cells circulating in the PB is still a matter of debate (16, 41). Moreover, it has been recently reported that, despite already “imprinted” in the early phases of solid tumor development, T cell dysfunctions are further amplified with disease progression and severity, observation that can be particularly relevant in the case of PDAC (42, 43). Of relevance, such dysfunctional state was only initially therapeutically reversible, then evolving in a fixed state (44). In perspective, it would be interesting to expand our analysis to other solid malignancies and hematological tumors characterized by severe intrinsic T cell defects, like CLL and acute myeloid leukemia (AML) (11, 36, 45, 46).

It has been recently described that patients with AML relapsing after hematopoietic stem cell transplantation have a higher proportion of early memory T cells, including T_SCM_, expressing multiple inhibitory receptors compared to patients achieving complete remissions (47). These evidences suggest that the exhaustion of specific T cell memory compartments could be critical to define response to the manufacturing procedures. Moreover, it is known that “quorum sensing” mechanisms between memory and naïve T cells culminate in the synchronization of T_N_ cell behavior to that of memory T cells, resulting in an accelerated differentiation at the transcriptional, metabolic and functional level (48). Consequently, the presence of dysfunctional antigen-experienced T cells may negatively shape the differentiation of T_N_, compromising the overall quality of CAR T cell products.

Finally, even though we cannot formally rule out a role of regulatory T cells (Tregs) in poor-performing CAR T cell products, culture in the presence IL-7, which has been reported to inhibit both Treg expansion and suppressive activity (49), and the clear effector signature characterizing our cell products, suggest that Tregs are not crucially involved in our setting.

Our data point out that additional strategies are required to customize fully functional CAR T cells in specific disease conditions. One possibility is to exploit allogeneic T cell sources, which have the advantage of being unaffected by prior treatments or by the tumor itself (33). Even though this option is limited by the risk of graft versus host reactions (GVHD), gene-editing approaches aimed at eliminating the endogenous TCR are currently becoming more common (8, 33). However, further preclinical investigation is still required to improve their safety profile, therefore imposing caution. An alternative strategy can be to deeper investigate and overcome initial T cell defects in the autologous setting. For example, it is possible to further optimize the manufacturing procedure by supplementing compounds known to expand T_SCM_, e.g., N-acetylcysteine (NAC), a reagent able to inhibit the metabolism of reactive oxygen species (24) or to pre-select definite T cell subsets as source material to get rid of more differentiated T cells. Directly inhibiting T cell exhaustion is another valuable option that can be achieved by combining CAR T cell therapy with checkpoint inhibitors (50, 51) or by additional genetic engineering of CAR T cell products (8). In particular, a recently proposed innovative strategy relies on CAR T cells over-expressing C-Jun, a transcription factor belonging to the AP-1 family, that resulted in resistance to exhaustion, enhanced *in vivo* expansion, reduced terminal differentiation and higher anti-tumor potency (52).

In this work, we also tried to identify informative efficacy biomarkers. By retrospectively analyzing CAR T cell-mediated anti-leukemia responses *in vivo*, we identified two cohorts of short- and long-term responders, characterized by relapses occurring at different interval times. Specific examination of these cohorts revealed that *in vivo* CAR T cell expansion represents a potent determinant of anti-leukemia efficacy, in accordance with clinical evidences (4, 10, 32). Moreover, in long-term responders, we observed CD8 prevalence soon after infusion, followed by predominance of CD4^+^ CAR T cells at later time points. These peculiar dynamics can be related to the need for rapid tumor de-bulking at early phases, provided by CD8^+^ CAR T cells, followed by the need for CD4^+^ CAR T cell help to achieve long-term anti-tumor control. Lastly, we observed that the frequency of T_SCM_ and CD8^+^ T cells in the final CAR T cell product positively correlated with T cell expansion *in vivo*, as opposed to the presence of more differentiated effector memory and CD57^+^ CAR T cells, which accounted as negative contributors. These observations indicate that such features need to be fostered during CAR T cell manufacturing and can be used as predictive biomarkers for the biological quality of CAR T cell products.

Overall, our work indicates that optimized manufacturing protocols can overcome initial T cell defects typical of cancer patients, resulting in CAR T cell products qualitatively equivalent to the ones generated from healthy donors. However, our results also highlight that the rescue of proper T cell functions cannot be achieved for specific tumor types, for which the development of further improved protocols and new strategies might be highly beneficial. Moreover, the crucial role of early memory T cells, especially T_SCM_, to achieve profound and durable anti-tumor responses *in vivo* was confirmed. Indeed, our data point out that next-generation CAR T cell manufacturing processes need to foster the enrichment of this T cell compartment, in order to significantly widen CAR T cell efficacy.

## Data Availability Statement

The datasets generated for this study are available on request to the corresponding author.

## Ethics Statement

This study was carried out in accordance with the recommendations of the IACUC: Institutional Animal Care and Use Committee (IACUC #646). The protocol received approval by the Italian Ministry of Health.

## Author Contributions

SA designed and performed experiments, analyzed data, and wrote the manuscript. LF and BC designed and performed the experiments. FD and MB performed experiments and analyzed the data and FD helped with the preparation of the figures. FG provided patient information. FC, CB, and AB actively contributed to the scientific discussion and manuscript revision. MC designed the study, analyzed and interpreted the data, wrote the manuscript, and acted as senior author of the study.

## Conflict of Interest

CB received research support from Intellia Therapeutics. AB is currently an employee of Novartis AG. His contribution to this work relates to the period 2016–17 when he was an employee of Innovative Immunotherapies Unit–Division of Immunology, Transplantation and Infectious Diseases—IRCCS San Raffaele Scientific Institute. The remaining authors declare that the research was conducted in the absence of any commercial or financial relationships that could be construed as a potential conflict of interest.
