# Supplementary material for: Next-Generation Manufacturing Protocols Enriching TSCM CAR T Cells Can Overcome Disease-Specific T Cell Defects in Cancer Patients
Source: Front Immunol. 2020 Jun 19;11:1217. doi: 10.3389/fimmu.2020.01217 (PMC7317024; doi:10.3389/fimmu.2020.01217)
Supplement: Supplementary file 1 [file Data_Sheet_1.pdf]

Supplementary Figure 1

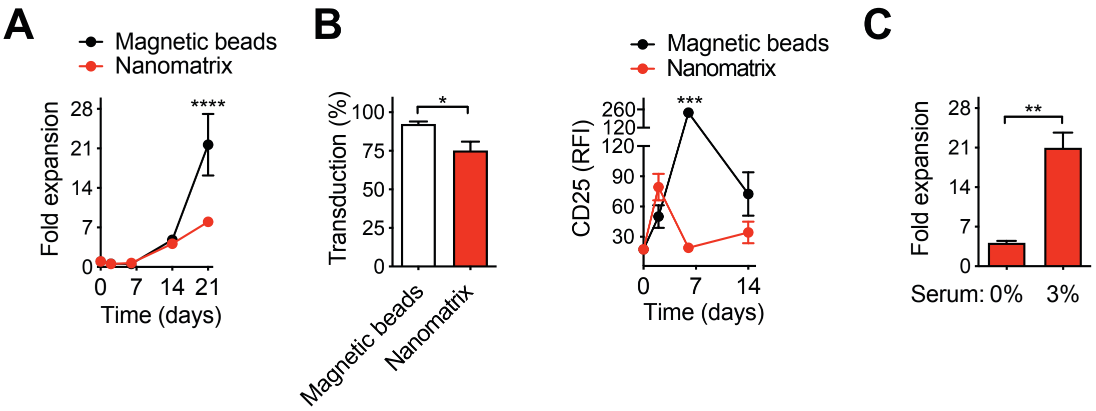

Supplementary Figure 1. Optimizing the conditions for CAR T cell generation.
A) Fold expansion of nanomatrix- and paramagnetic beads-generated CD19.BBz CAR T cells in the absence of fetal bovine serum (FBS) in the culture medium (n=3). **B)** Transduction efficiency at day 8 (left) and CD25 expression kinetic during culture (right) in the absence of FBS in the medium (n=3 for paramagnetic beads and n=6 for nanomatrix). **C)** Fold expansion of nanomatrix-generated CD19.BBz CAR T cells with or without 3% FBS in the culture medium (n=6). Data are reported as the result of mean ±SEM and paired t-test and two-way ANOVA statistical analyses are reported when statistically significant (*p<0.01; **p<0.01; *** p<0.001; ****p<0.0001).

Supplementary Figure 2

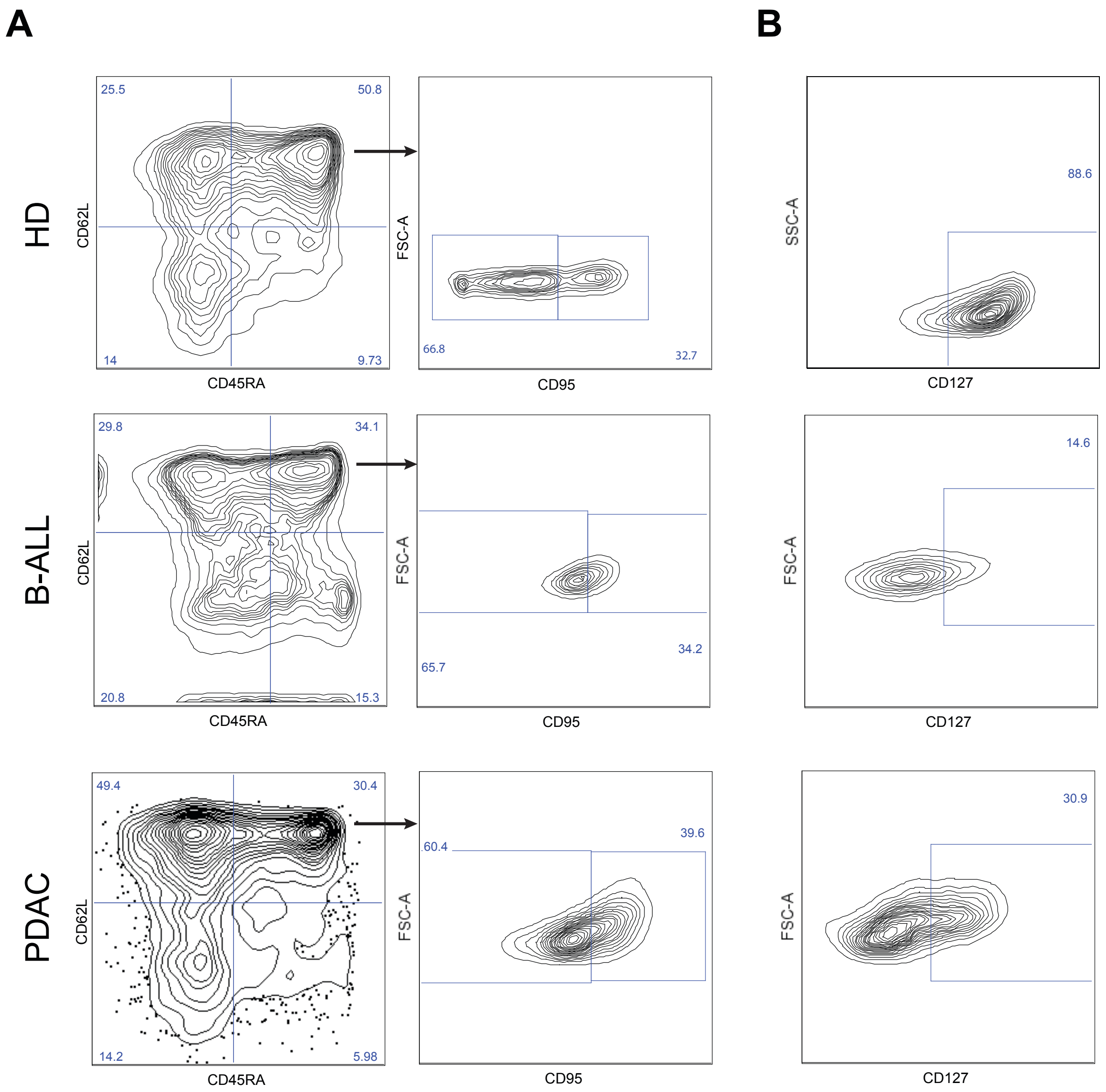

Supplementary Figure 2. T cells derived from patient samples are more differentiated compared to healthy donors prior T-cell stimulation.
Representative flow-cytometric dot plots depicting memory phenotype (**A**) and CD127 expression (**B**) of T cells retrieved from HD, B-ALL and PDAC patients before activation. T_N: $CD45RA^+CD62L^+CD95^-$; T_{SCM}: $CD45RA^+CD62L^+CD95^+$; T_{CM}: $CD45RA^-CD62L^+$; T_{EM}: $CD45RA^-CD62L^-$; T_{EMRA}: $CD45RA^+CD62L^-$.

Supplementary Figure 3

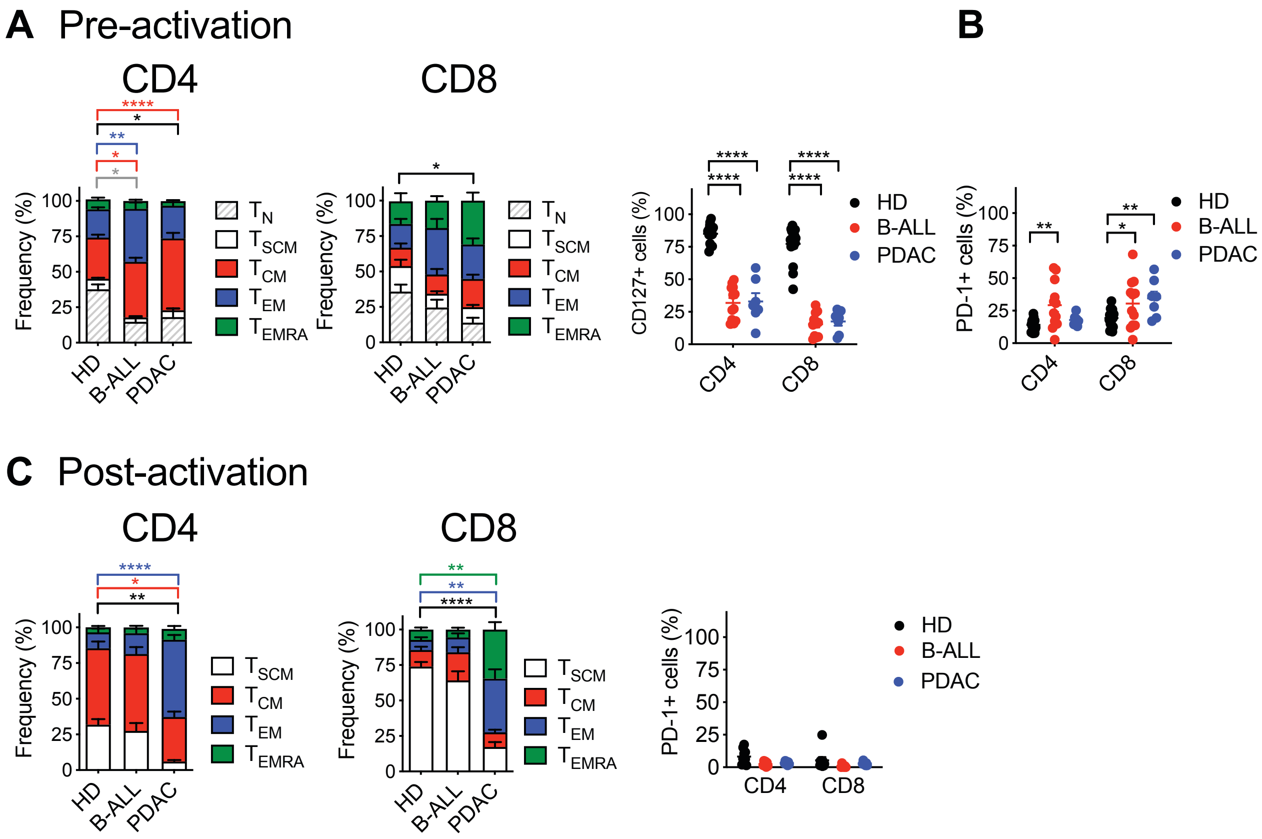

Supplementary Figure 3. TransAct optimized protocol for CAR T cell production is able to recover disease-specific CAR T cell effector functions of both CD4 and CD8 compartments. Phenotypical characterization of $CD4^+$ and $CD8^+$ T cells derived from the peripheral blood of healthy donors (HD) and patients suffering from B-cell acute lymphoblastic leukemia (B-ALL)- and pancreatic ductal adenocarcinoma (PDAC) before (**A,B**) and after (**C**) T-cell stimulation. **A)** Left panel: T-cell memory compartments (n=18 for HD; n=12 for B-ALL; n=7 for PDAC, T_N: $CD45RA^+CD62L^+CD95^-$; T_{SCM}: $CD45RA^+CD62L^+CD95^+$; T_{CM}: $CD45RA^-CD62L^+$; T_{EM}: $CD45RA^-CD62L^-$; T_{EMRA}: $CD45RA^+CD62L^-$). Right panel: IL-7Rα (CD127) expression (n=16 for HD; n=12 for B-ALL; n=7 for PDAC). **B)** PD-1 expression (n=19 for HD; n=12 for B-ALL; n=7 for PDAC). **C)** T-cell memory compartments and PD-1 expression (n=12 for HD; n=7 for B-ALL; n=7 for PDAC, T_{SCM}: $CD45RA^+CD62L^+$; T_{CM}: $CD45RA^-CD62L^+$; T_{EM}: $CD45RA^-CD62L^-$; T_{EMRA}: $CD45RA^+CD62L^-$). Data are reported as the result of mean ±SEM and paired t-test and two-way ANOVA statistical analyses are reported when statistically significant (*$p<0.05$, **$p<0.01$, ****$p<0.0001$).

Supplementary Figure 4

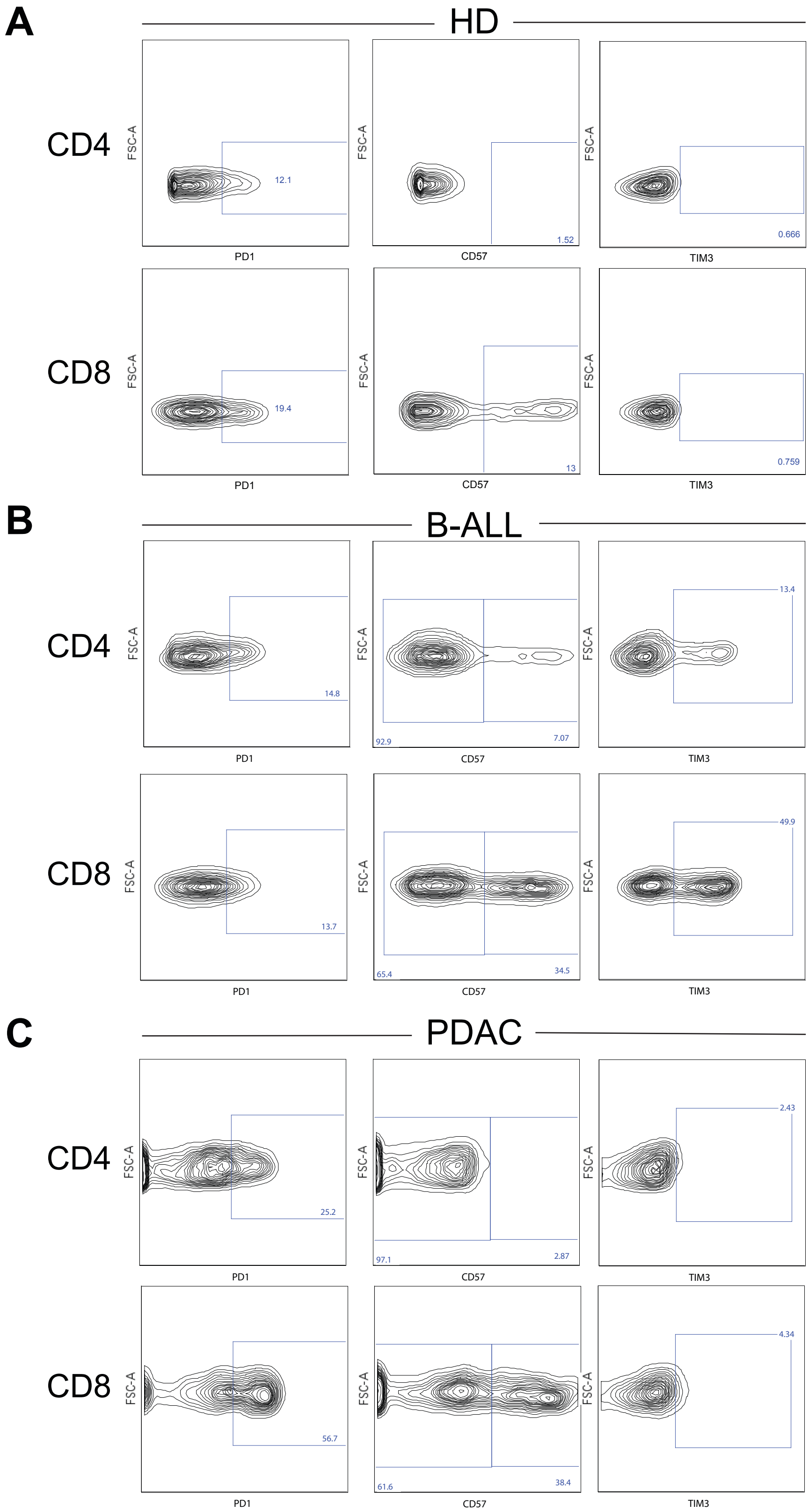

Supplementary Figure 4. T cells derived from patient samples are more exhausted compared to healthy donors prior T-cell stimulation.

Representative flow-cytometric dot plots depicting exhaustion (PD-1, TIM-3) and senescence (CD57) markers in the CD4 and CD8 T-cell compartments of HD (**A**), B-ALL (**B**) and PDAC (**C**) patient samples before activation.

Supplementary Figure 5

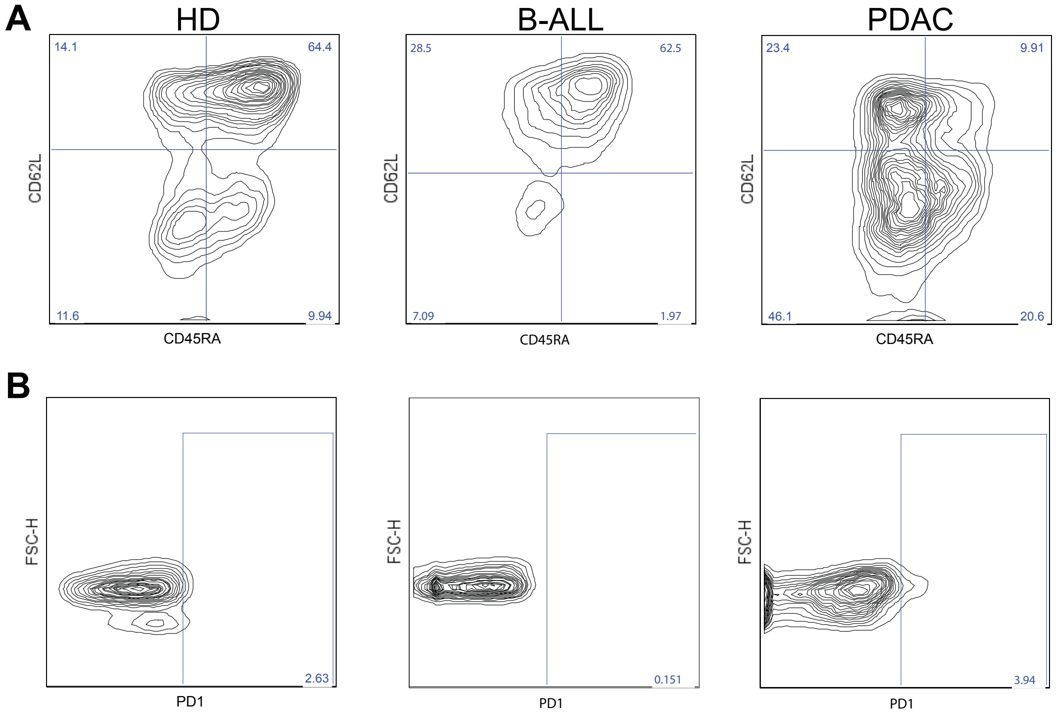

Supplementary Figure 5. CAR T cells derived from PDAC, but not B-ALL patient samples retain a more differentiated and exhausted phenotype compared to healthy donors after optimized manufacturing.

Representative flow-cytometric dot plots depicting memory phenotype (**A**) and PD-1 expression (**B**) of T cells generated from HD, B-ALL and PDAC patients. T_{SCM}: $CD45RA^{+}CD62L^{+}$; T_{CM}: $CD45RA^{-}CD62L^{+}$; T_{EM}: $CD45RA^{-}CD62L^{-}$; T_{EMRA}: $CD45RA^{+}CD62L^{-}$.

Supplementary Figure 6

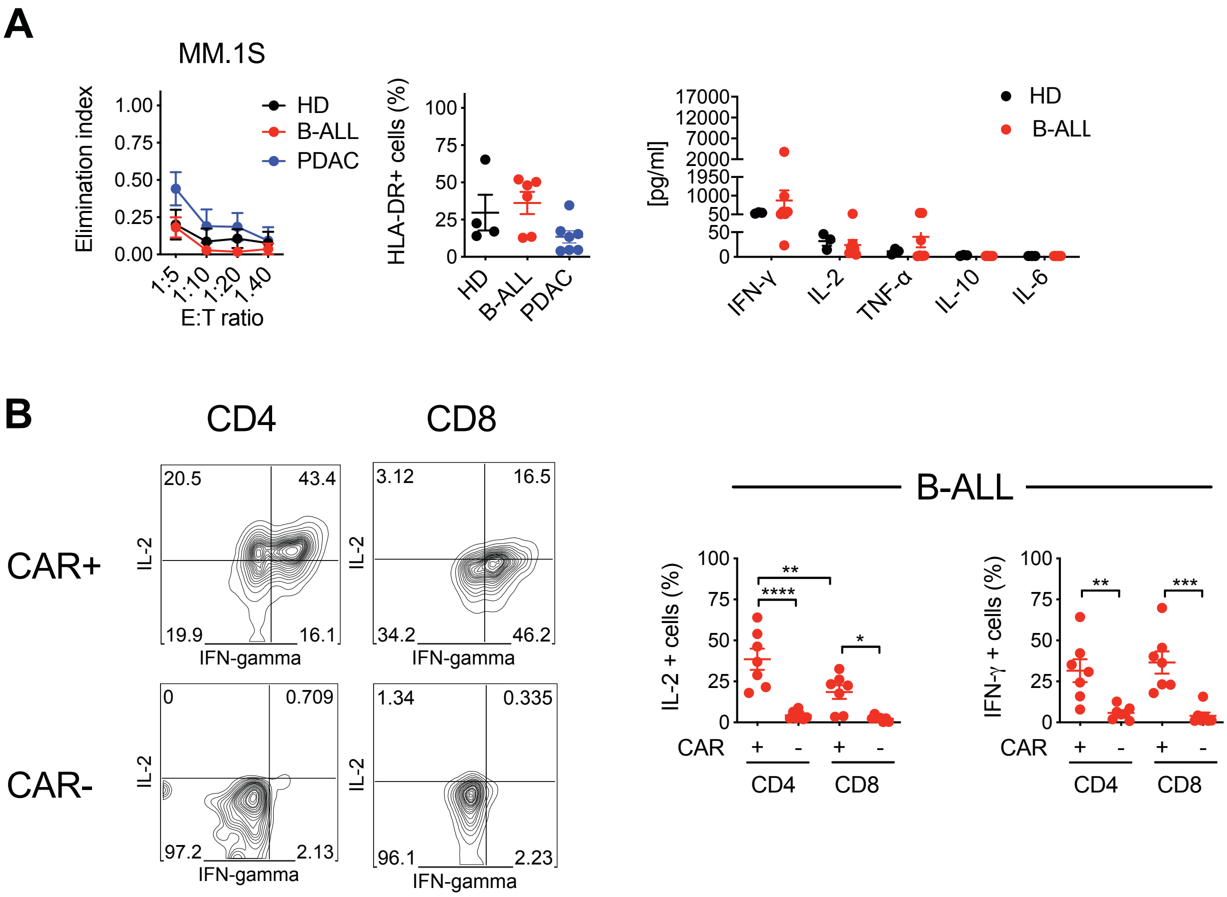

Supplementary Figure 6. TransAct generated CD19.BBz CAR T cells derived by healthy donors as well as by tumor patients display *in vitro* effector functions specifically for CD19^{+} targets.

A) Co-culture assays performed by co-incubating HD- and patient-derived CD19.BBz CAR T cells with CD19^{-} MM-1S cells for 4 days at different E:T ratios (n=3 for HD; n=6 for B-ALL; n=7 for PDAC). Left panel: killing activity expressed as elimination index. Middle panel: CAR T cell activation, expressed as HLA-DR upregulation at the 1:10 E:T ratio (n=3 for HD; n=6 for B-ALL; n=7 for PDAC). Right panel: quantification of pro-inflammatory cytokine production after 24h at the 1:10 E:T ratio (n=3 for HD; n=6 for B-ALL). **B)** Intracellular staining depicting IFN-γ and IL-2 production by CD4 and CD8 B-ALL-derived CD19.BBz CAR T cells (positive and negative fractions) after 24h stimulation with CD19^{+} targets (BV-173 and NALM-6). Left panel: representative dot plots. Right panel: mean ± SEM (n=7). Paired t-test and two-way ANOVA statistical analyses are reported when statistically significant (*p<0.05, **p<0.01, ***p<0.001, ****p<0.0001).
